# Effect of Remote Ischemic Preconditioning on Perioperative Cardiac Events in Patients Undergoing Elective Percutaneous Coronary Intervention: A Meta-Analysis of 16 Randomized Trials

**DOI:** 10.1155/2017/6907167

**Published:** 2017-09-14

**Authors:** Xiangming Wang, Na Kong, Chuanwei Zhou, Deeraj Mungun, Zakaria Iyan, Yan Guo, Zhijian Yang

**Affiliations:** ^1^Department of Geriatric Cardiology, The First Affiliated Hospital of Nanjing Medical University, Nanjing, China; ^2^Reproductive Medicine Center, The Affiliated Drum Tower Hospital of Nanjing University, Nanjing, China; ^3^Department of Cardiology, The First Affiliated Hospital of Nanjing Medical University, Nanjing, China

## Abstract

**Background:**

The main objective of this meta-analysis was to investigate whether remote ischemic preconditioning (RIPC) reduces cardiac and renal events in patients undergoing elective cardiovascular interventions.

**Methods and Results:**

We systematically searched articles published from 2006 to 2016 in PubMed, EMBASE, Web of Science, Cochrane Library, and Google Scholar. Odds ratios (ORs) with 95% confidence intervals (CIs) were used as the effect index for dichotomous variables. The standardized mean differences (SMDs) with 95% CIs were calculated as the pooled continuous effect. Sixteen RCTs of 2435 patients undergoing elective PCI were selected. Compared with control group, RIPC could significantly reduce the incidence of perioperative myocardial infarction (OR = 0.64; 95% CI: 0.48–0.86; *P* = 0.003) and acute kidney injury (OR = 0.56; 95% CI: 0.322–0.99; *P* = 0.049). Metaregression analysis showed that the reduction of PMI by RIPC was enhanced for CAD patients with multivessel disease (coef.: −0.05 [−0.09; −0.01], *P* = 0.022). There were no differences in the changes of cTnI (*P* = 0.934) and CRP (*P* = 0.075) in two groups.

**Conclusion:**

Our meta-analysis of RCTs demonstrated that RIPC can provide cardiac and renal protection for patients undergoing elective PCI, while no beneficial effect on reducing the levels of cTnI and CRP after PCI was reported.

## 1. Introduction

Percutaneous coronary intervention (PCI) is one of the most important treatments for coronary artery disease. In acute myocardial infarction, timely myocardial reperfusion therapy, such as PCI, CABG, and Thrombolysis, is an effective method to limit the myocardial infarct area, attenuate clinical symptoms, and improve the clinical prognosis. However, reperfusion may induce further damage to the myocardium itself [1, 2]. Myocardial ischemia-reperfusion injury (MIRI) is a common pathophysiological process that poses a serious threat to patients' health. Many studies have shown that elevated levels of cTnI after PCI are associated with a poor prognosis in patients with coronary artery disease [3–7]. In recent years, many clinical studies have confirmed that RIPC provides effective myocardial protection in patients undergoing PCI, and RIPC is an important method to prevent MIRI.

While RIPC's cardioprotective effect has been seen in patients undergoing selective PCI, many clinical trials have examined whether RIPC has a protective effect on these patients [8–11]. Unfortunately, studies on the protective effects of RIPC in PCI patients are limited, and the results are controversial and contradictory because not all of the trials have observed the beneficial effects of RIPC. D'Ascenzo et al.'s meta-analysis showed that RIPC could reduce the incidence of PCI-related myocardial infarction, but PCI did not affect CRP after the procedure [12]. However, it is important to note that there were fewer studies included in the meta-analysis (5 studies with 731 subjects). A new meta-analysis reported by Pei et al. [13] in 2014, which included 11 studies and a total of 2,301 patients, demonstrated that RIPC could provide heart and kidney protection by reducing the incidence of MI and AKI in patients with selective PCI. In the past two years (2014–2016), new randomized controlled trials (RCTs) have been published; these findings suggest that the incidence of MI after PCI, the incidence of MACCE at 6 months after PCI, and the effect of PCI on renal function are different. These RCTs were not included in previous meta-analyses, and the role of RIPC in patients undergoing PCI needed to be reassessed. Thus, we conducted a comprehensive meta-analysis to study whether RIPC (compared with the controls) provided myocardial and renal protection for patients undergoing selective PCI.

## 2. Materials and Methods

### 2.1. Search Strategy

We performed this meta-analysis according to the PRISMA (Preferred Reporting Items for Systematic reviews and Meta-Analyses) statement [14] and the Cochrane Handbook for Systematic Reviews [15]. We systematically searched articles published from 2006 to 2016 in the following databases: PubMed, EMBASE, Web of Science, Cochrane Library, and Google Scholar. Our research was last updated on December 30, 2016. The following search phrases or keywords were used: “remote ischemic preconditioning,” “ischemic preconditioning,” “limb ischemic preconditioning,” “elective percutaneous coronary intervention,” “myocardial injury,” and “cardioprotection.”

### 2.2. Inclusion and Exclusion Criteria

The inclusion criteria were as follows: (1) RCTs published in English, (2) studies that involved patients undergoing elective PCI, (3) studies that reported the incidence of perioperative myocardial infarction or troponin levels after PCI or renal injury as endpoints, and (4) RIPC intervention regardless of the duration or number of cycles. The exclusion criteria were as follows: (1) repeated published literature, (2) trials that used RIPC in combination with another concomitant intervention, (3) incomplete original research data, (4) studies that included patients with ST-segment elevation myocardial infarction, (5) animal studies, and (6) nonrandomized clinical trials.

### 2.3. Data Extraction

Two researchers (WXM and ZI) independently screened the titles, abstracts, and the full articles as needed, and then they determined whether the studies met the inclusion criteria. When the researchers did not agree, the problems were resolved through a discussion or by a third-party reviewer (DM or KN) to make a determination. The researchers extracted the data from all of the qualifying articles and assessed the bias risk. If necessary, we directly contacted the original author for information. The main data extracted included basic research information (including the title, the first author, and the publication year), research characteristics (including sample size, age, gender, diabetes mellitus, hypertension, heart failure, drugs, and vascular characteristics,), outcome indicators and the results of measurement data (i.e., the incidence of PMI, the incidence of AKI and MACCE, and serum or plasma cTns levels), and the key elements of bias risk assessment. We converted some of the original text in the “median and range” of the results of the indicators to “mean and standard deviation” through the O'Rourke method. The quality of the studies was assessed using Jadad et al.'s scoring system: randomization, blinding, and providing an explanation for withdrawals and dropouts [16]. Studies with a Jadad et al.'s score of greater than or equal to 3 points were considered to be high-quality trials.

### 2.4. Statistical Methods

The meta-analysis was performed using Stata software (version 12.1; StataCorp LP, College Station, TX, USA). Odds ratios (ORs) with 95% confidence intervals (CIs) were used as the effect index for dichotomous variables, such as the incidence of PMI and the incidence of AKI and MACCEs. The standardized mean differences (SMDs) with 95% CIs were calculated as the pooled continuous effect. Heterogeneity among studies was assessed by means of the chi-square-based *Q* test and the *I*^2^ index [17]. *I*^2^ > 50% or *P* < 0.05 indicated evidence of heterogeneity. When *I*^2^ < 50%, studies were considered to be heterogeneous, and fixed-effects models were used for analysis, whereas if heterogeneity was significant (*I*^2^ value ≥ 50% or *P* < 0.05), random-effects models were selected [18, 19]. To further investigate the possible sources of heterogeneity, subgroup analysis or metaregression analysis was performed. Forest plots were drawn to evaluate the effects of RIPC on every outcome. Sensitivity analyses were performed to assess the stability of the results. Publication bias was assessed using Begg's funnel plot and Egger's linear regression tests. A *P* value <0.05 indicated a statistically significant difference.

## 3. Results

### 3.1. Literature Search Results

**Figure 1:**
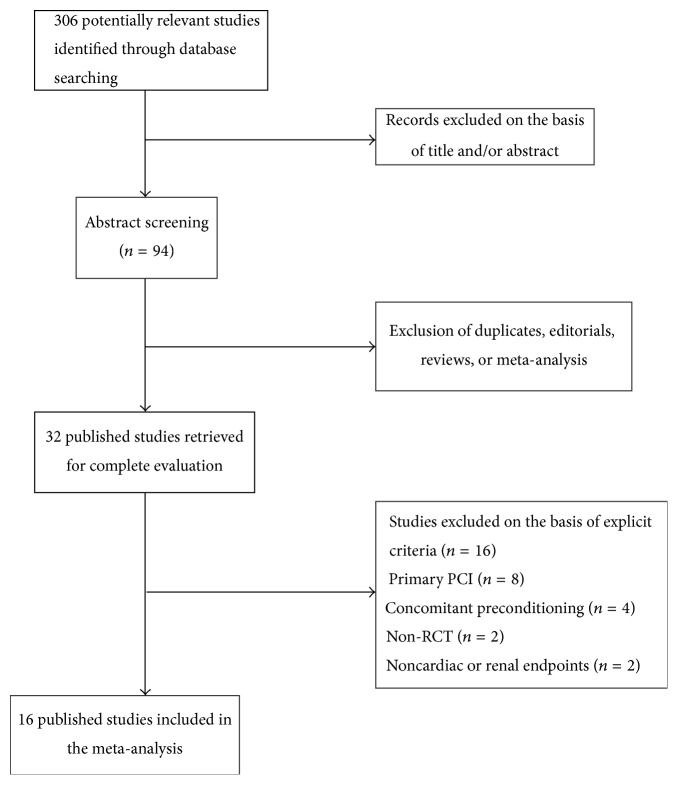
Flow chart of the studies identified with criteria for inclusion and exclusion.

A total of 306 citations were initially screened after searching the databases. We reviewed the article titles and extracts, and then we excluded the studies that did not meet the inclusion standards; the full texts of 31 trials were further evaluated. Of these, 15 trials were excluded: 8 due to study patients undergoing emergency PCI [20–27], 2 because endpoints were not evaluated, 3 because concomitant preconditioning treatments were used [28–30], and 2 because they were not RCTs. Lavi et al.'s trial [31] was divided into two independent studies because of the different preconditioning protocols (expressed as Lavi I and Lavi II). Finally, a total of 16 randomized controlled trials were included in the meta-analysis [8–11, 23, 31–40], with the literature screening process and results shown in Figure 1.

### 3.2. Study Characteristics

A total of 2,435 patients were enrolled (from 11 countries) in the included studies, with 1,215 patients randomized to the RIPC group and 1,220 patients to the control group. RIPC was performed by inflating a blood pressure cuff that was placed on the upper limb or leg to 200 mmHg or above the basic systolic pressure over 10 mmHg. The ischemic-reperfusion protocol [cycles × *I*/*R*] was 3 × 5 min/5 min in 7 studies [8, 9, 11, 35, 36, 38, 39], 4 × 5 min/5 min in 1 study [32], 2 × 5 min/5 min in 1 study [34], 3 × 3 min/3 min in 3 studies [10, 34, 41], 1 × 5 min/5 min in 3 studies [31, 40], and 4 × 30 sec/30 sec in 1 study [23]. Among these trials, 11 studies reported the incidence of PMI [8–10, 31, 33, 35–38, 40], and 7 studies reported the incidence of AKI [10, 23, 31, 32, 35, 38]. There were 15 studies that reported the levels of myocardial injury biomarkers after PCI, with 10 trials using troponin I or T and 5 trials using CK-MB. The baseline characteristics were comparable between the RIPC group and the control group; their median age was 65.15 years and 69.2% of the patients were males. The percentages of patients with diabetes, hypertension, and dyslipidemia were 51.01%, 72.70%, and 63.05%, respectively. Of the patients, 61.81% were treated with angiotensin-converting enzyme inhibitors and 70.4% with beta-blockers; 31.1% of the patients presented with multivessel disease and 38.56% with a type C lesion. There were no statistically significant differences in patients' gender, their ages, and the preoperative eGFR levels between the two groups. The patients' baseline characteristics and the trial design of all of the included randomized trials are shown in Tables 1 and 2. The quality of the included studies was assessed using Jadad et al.'s score as shown in Table 3. In terms of research quality, 12 studies had a Jadad et al.'s score ≥3 points, and 4 studies had Jadad et al.'s scores of <3 points.

### 3.3. Effects of RIPC on the Incidence of PMI

**Figure 2:**
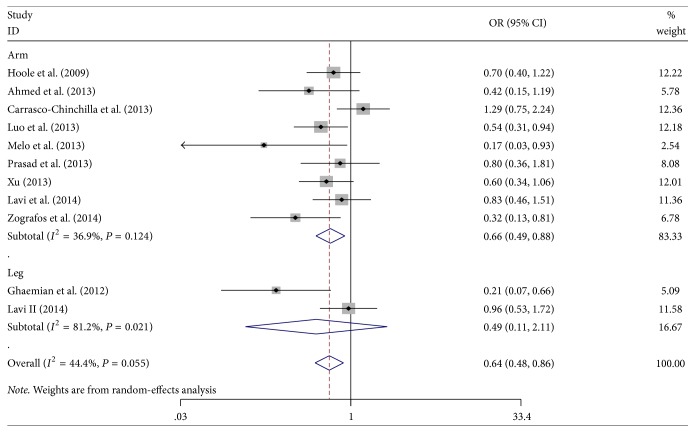
Forest plot for the incidence of perioperative myocardial infarction (PMI). RIPC: remote ischemic preconditioning; OR: odds ratio.

**Figure 3:**
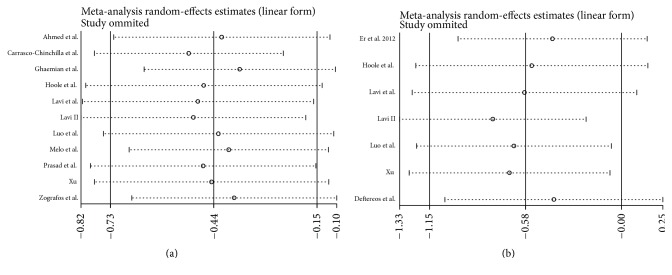
Sensitivity analysis of the effect of RIPC on PMI (a) and AKI (b).

In 16 studies, 11 studies reported the incidence of PMI in patients. There was moderate heterogeneity in the 11 studies (*P* = 0.101, *I*^2^ = 44.4%), so we performed a meta-analysis using a random-effects model. The meta-analysis showed that the incidence of PMI in the RIPC group was significantly lower than that in the control group (OR = 0.64; 95% CI: 0.48–0.86; *P* = 0.003). The RIPC of the upper arm significantly prevented PMI (OR = 0.66; 95% CI: 0.49–0.88; *P* = 0.005; Figure 2); however, the incidence of PMI was not reduced by RIPC of the lower limb in patients (OR = 0.491; 95% CI: 0.11–2.11; *P* = 0.339). The leave-one-out sensitivity analysis, which removed individual studies one by one, showed that no single study significantly altered the overall effect of RIPC on reducing PMI (all *P* < 0.05, Figure 3(a)).

### 3.4. Effect of Remote Ischemic Preconditioning on the Incidence of AKI

**Figure 4:**
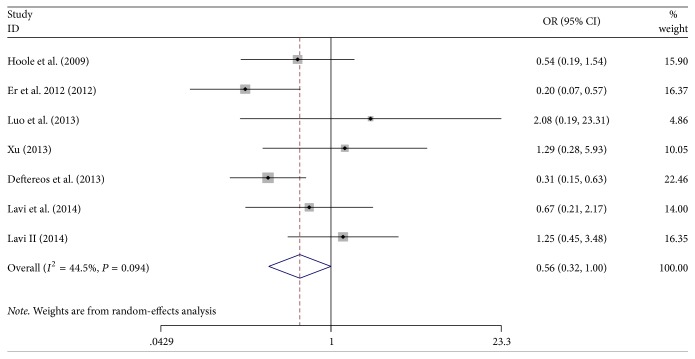
Forest plot for the incidence of acute kidney injury (AKI). RIPC: remote ischemic preconditioning; OR: odds ratio.

AKI after PCI was reported in 1,378 study subjects, and the overall incidence was 9.14% (45/698 in the RIPC group and 81/680 in the control group). This group of studies showed moderate heterogeneity (*P* = 0.094, *I*^2^ = 44.5%). The incidence of AKI in the remote preconditioned patients was significantly lower than that in the control groups (OR = 0.56; 95% CI: 0.32–0.99; *P* = 0.049; Figure 4). Sensitivity analysis revealed that our results were reliable and robust by excluding each included trial one at a time (all *P* < 0.05; Figure 3(b)).

### 3.5. cTnI Concentrations after PCI

**Figure 5:**
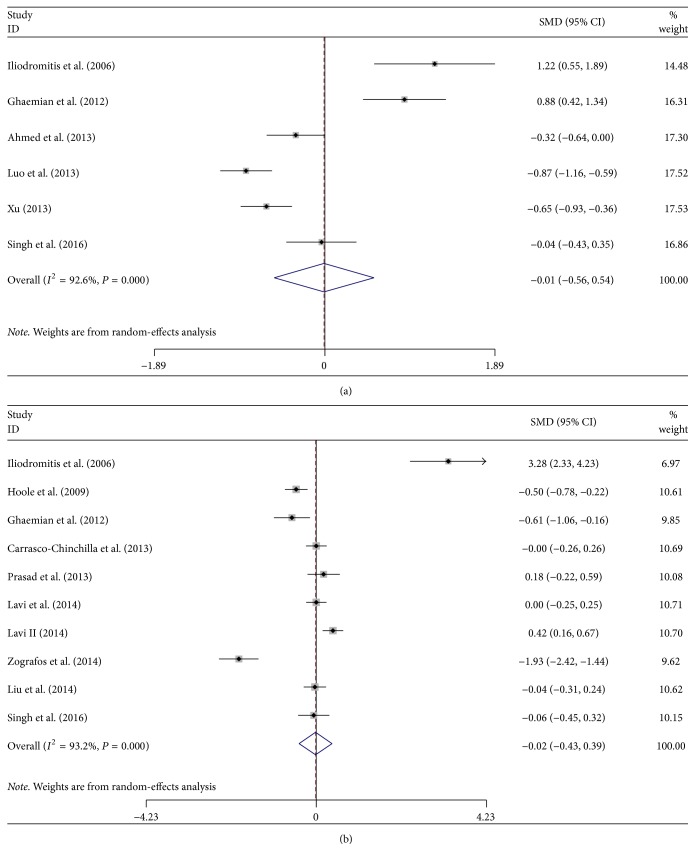
Forest plot for myocardial biomarkers expressed as SMD within 12 h (a) and 24 h (b) after PCI. SMD: standardized mean difference.

Data about the cTnI concentrations after PCI were available in 13 of the trials. There were 10 studies that reported the cTnI levels at 24 h after PCI and 6 studies at 12 h after PCI. For the cTnI concentration at 12 h postoperatively, there was no significant difference between the RIPC group and the control group (SMD −0.11; 95% CI: −0.48–0.27; *P* = 0.585) with significant heterogeneity (*P* < 0.001, *I*^2^ = 92.6%; Figure 5(a)). Similarly, for the cTnI concentrations at 24 h postoperatively, there was also no significant difference between the RIPC group and the control group (SMD: −0.02; 95% CI: −0.43–0.39; *P* = 0.934; Figure 5(b)) with significant heterogeneity (*P* < 0.001, *I*^2^ = 93.2%).

### 3.6. Levels of CRP after PCI

**Figure 6:**
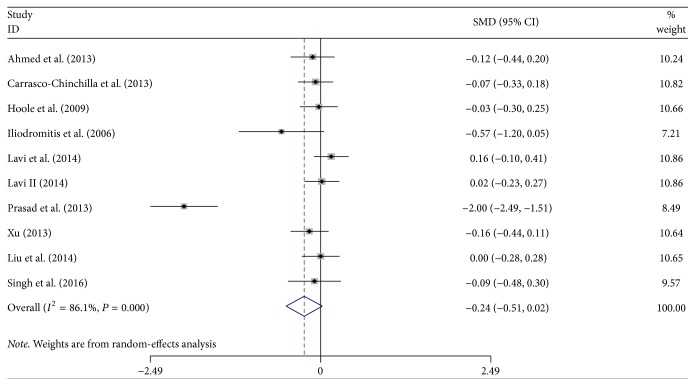
Forest plot for CRP as SMD after PCI. CRP: C-reactive protein; SMD: standardized mean difference.

There were 10 studies that reported CRP levels at 12–24 h after PCI. The studies about CRP had significant heterogeneity (*χ*^2^ = 0.152; *P* < 0.001; *I*^2^ = 86.1%), so we performed a meta-analysis with random-effects models. The results showed that there were no significant differences in the CRP concentrations after PCI between the two groups (SMD: −0.24; 95% CI: −0.51–0.024; *P* = 0.075; Figure 6).

### 3.7. Publication Bias

**Figure 7:**
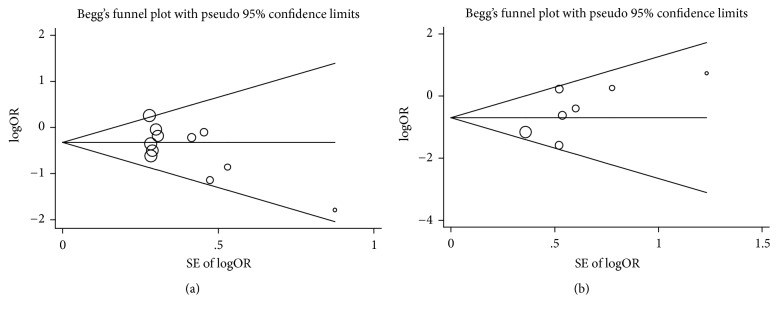
Begg's funnel plot for publication bias test. (a) The incidence of PMI and (b) the incidence of AKI.

Publication bias was evaluated by Begg's funnel plot and Egger's test (see Supplementary Figure  2 in Supplementary Material available online at https://doi.org/10.1155/2017/6907167). We found that there was no significant publication bias in the studies about the incidence of PMI (*P* = 0.139, Begg's test; *P* = 0.065, Egger's test; Figure 5) and the incidence of AKI (*P* = 0.176, Begg's test; *P* = 0.116, Egger's test). The shapes of the funnel plots seemed symmetrical for the levels of cTnT at 24 h after PCI (*P* = 0.325); this finding was also supported by Egger's test (*P* = 0.853). However, the results revealed that potential publication biases existed in the levels of cTnI 12 h after PCI (*P* = 0.039, Begg's test; *P* = 0.006, Egger's test) and in the CRP levels (*P* = 0.009, Begg's test; *P* = 0.022, Egger's test). All of Begg's funnel plots for the publication bias tests are presented in Figure 7 and Supplementary Figure  1.

### 3.8. Metaregression Analyses

**Figure 8:**
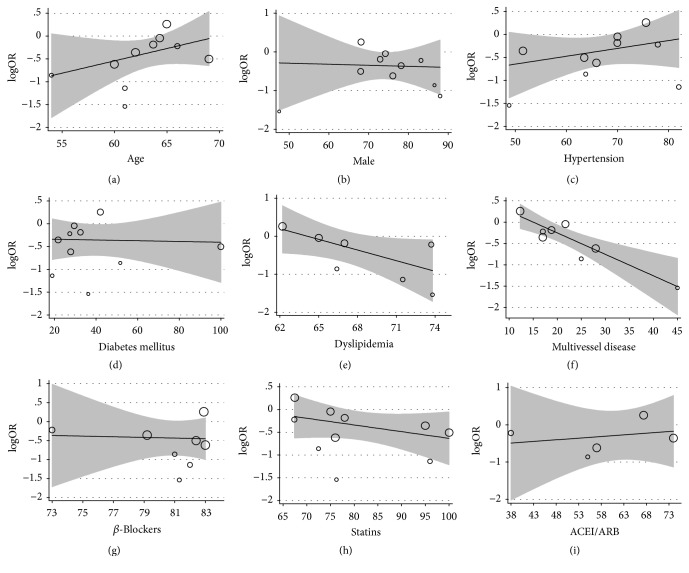
Metaregression results of reduction of PMI by RIPC. Metaregression of age (a), percentage of males (b), percentage of hypertension (c), percentage of diabetes mellitus (DM) (d), percentage of dyslipidemia (e), percentage of multivessel disease (f), percentage of *β*-blockers used (g), percentage of statins used (h), and percentage of ACEI/ARB used (i).

Random-effects metaregression analysis showed that RIPC's protective effect was enhanced for patients with multivessel disease (coef.: –0.05 [–0.09; –0.01], *P* = 0.022). We did not find any significant relationship between the incidence of PMI and other confounding factors, such as age (coef.: 0.059 [−0.03; 0.15], *P* = 0.118), the percentage of patients being male (coef.: 0.002 [−0.039; 0.044], *P* = 0.916), the percentage of diabetes mellitus (coef.: −0.003 [−0.015; 0.015], *P* = 0.960), the percentage of hypertension (coef.: 0.017 [−0.019; 0.054], *P* = 0.320), the percentage of dyslipidemia (coef.: 0.093 [−0.195; 0.008], *P* = 0.063), the use of beta-blockers (coef.: −0.021 [−0.179; 0.138], *P* = 0.759), the use of statins (coef.: −0.05 [−0.15; 0.05], *P* = 0.35), and the use of angiotensin-converting enzyme inhibitors (coef.: 0.007 [−0.049; 0.064], *P* = 0.697). The results of the metaregression analysis are shown in Figure 8.

## 4. Discussion

In the present meta-analysis of 16 randomized trials that enrolled 2,435 adult patients who underwent elective PCI, we evaluated whether remote ischemic preconditioning can offer a protective effect by reducing cardiac and renal events.

Coronary artery disease (CAD) is the most common cause of death in developed and some developing countries. Coronary revascularization with medical therapy and lifestyle alteration constitutes the modern management of patients with significant CAD. In acute myocardial infarction, timely myocardial reperfusion therapy, such as PCI, CABG, and Thrombolysis, is an effective method to limit the myocardial infarct area, attenuate clinical symptoms, and improve the clinical prognosis. However, a large number of studies have shown that reperfusion can lead to further damage to the heart itself [1, 2]. Myocardial ischemia-reperfusion injury (MIRI) is a common pathophysiological process, and it is a serious threat to patients' health.

Coronary revascularization by elective percutaneous coronary intervention (PCI) is the principal intervention in patients with stable CAD and acute coronary syndrome. Even though technical advances in PCI over the past two decades have resulted in a safe procedure with minimal complications, in several patients, the procedure is complicated by periprocedural injury, which can be detected by elevated values of myocardial necrosis biomarkers. Several studies have reported that periprocedural injury is associated with a worse prognosis [42, 43]. A high level of cTnI in patients undergoing PCI was an independent predictor of composite endpoint events (death, myocardial infarction, and revascularization) within 1 year [3–7]. With the progress of coronary heart disease intervention in the past two decades, surgical complications and long-term efficacy have been significantly improved; however, periprocedural myocardial infarction is still very common. Therefore, great efforts have been focused on the prevention of periprocedural complications in recent years.

Ischemic preconditioning (IPC) was first described in a study by Murray et al. in 1986 [44]. The cardioprotective effects of RIPC are also being explored in patients undergoing elective PCI. In 2009, Hoole et al. [10] extended the concept of RIPC to show that RIPC—induced by 3 5-minute blood pressure cuff inflations to 200 mmHg around the upper arm, interspersed with 5 minutes of reperfusion, before the patient's arrival in the catheterization laboratory for stenting—significantly reduced median troponin I concentrations at 24 h (0.06 ng/mL) compared with the control patients (0.16 ng/mL; *P* < 0.04). In the past, numerous clinical trials examined whether RIPC has a protective effect on PCI patients [8–11]; however, the studies regarding RIPC's protective effect in patients undergoing PCI were limited, and the results were controversial and contradictory.

The evidence from the present meta-analysis showed that RIPC can provide myocardial protection in patients undergoing PCI. In previous studies, RIPC has been shown to prevent myocardial ischemia-reperfusion injury in patients undergoing cardiovascular interventional procedures, and a number of meta-analyses showed that RIPC reduced myocardial injury markers and reduced perioperative myocardial infarction. The meta-analyses by D'Ascenzo et al. [12] and Pei et al. [13], which evaluated the effect of RIPC in the patients undergoing cardiac interventions, showed that the incidence of PMI was reduced by RIPC. Our latest study, which includes nearly two years of inclusion in a meta-analysis, also shows that RIPC is effective in preventing PMI, and it is consistent with previous meta-analyses. In the subgroup analysis, we compared preconditioning of upper and lower extremities and found that RIPC of the upper extremities had a statistically significant effect on protecting PMI; however, RIPC with lower limb preconditioning cannot effectively reduce the incidence of PMI, which is inconsistent with the results of a meta-analysis by D'Ascenzo et al. In our analysis, there was greater heterogeneity in the clinical studies of limb preconditioning, particularly in Lavi et al.'s limb preconditioning procedure, which used only a 5-minute ischemia-reperfusion cycle, and the strength of the preconditioning could lead to a change in outcome. Previous studies have shown that the intensity of distal limb ischemia and protective effects are closely related [12]. The different mechanisms of RIPC between the upper arm and the lower limb remain unclear. There are still a limited number of studies to evaluate whether RIPC with the upper arm is different from RIPC with the lower limb in relation to the protective effects for PMI. Therefore, future research is needed to compare these two types of RIPC to determine whether they exhibit different capacities for cardiac protection.

To further investigate the sources of heterogeneity, we performed metaregression analysis. We did not find any significant relationship between the incidence of PMI and other confounding factors, such as age, the percentage of male patients, the percentage of hypertension patients, the percentage of diabetes mellitus patients, the percentage of dyslipidemia patients, and their medication. Surprisingly, the reduction of PMI by RIPC was enhanced for patients with multivessel disease. These observations are consistent with previous published reports of RIPC in patients with diffuse coronary artery disease who underwent CABG surgery [45]. Our results may explain why some studies with a low sample size and a relatively low risk (a proportion of low diabetes mellitus and multivessel disease) failed to observe the effect of RIPC on PMI. In other words, the more the disease is diffused, the more significant RIPC's reduction of the effect of PMI is.

Many clinical observations have found that increased levels of myocardial injury markers, such as troponin T, troponin I, and CK-MB, are associated with adverse long-term prognosis after elective percutaneous coronary intervention [41, 46, 47]. In the meta-analysis by Niu et al. [48], it was found that RIPC can reduce myocardial injury markers after PCI release; this protective effect was more obvious in STEMI patients, while in the elective PCI patients, RIPC cannot reduce myocardial injury markers, and the heterogeneity of the included studies was larger. To our knowledge, this is the first meta-analysis to explore whether RIPC could reduce the release of myocardial markers after PCI in patients with elective PCI. The results showed that RIPC was unable to reduce the concentration of cTnI at 12 h and 24 h after elective PCI (for 12 h, SMD: −0.11, 95% CI: −0.48–0.27, *P* = 0.585; for 24 h, SMD: −0.02, 95% CI: −0.43–0.39, *P* = 0.934), and there was a high degree of heterogeneity in the included studies (*I*^2^ = 93.2%). We considered that the possible reason that there were no effective results in RIPC reduction of cTnI is because myocardial injury during elective PCI is relatively minimal compared with that during acute myocardial infarction, which is mainly due to coronary artery side branch loss and distal embolization during balloon inflation or stent implantation [49, 50].

AKI is a serious postoperation complication in patients with cardiac and vascular interventions. Patients with postoperative acute kidney injury have significantly higher morbidity and mortality [44]. To date, whether RIPC can or cannot protect against kidney injury in patients undergoing percutaneous coronary intervention is still a controversial issue. Li et al. performed a meta-analysis in which they found that RIPC can reduce contrast-induced AKI in patients undergoing PCI/CAG [51]. Similarly, Alreja et al.'s meta-analysis [52] revealed that RIPC can also significantly reduce AKI incidence in patients undergoing cardiac or vascular interventions, but there was high heterogeneity among the 26 trials they analyzed. Conversely, D'Ascenzo et al. [12] and Brevoord et al. [53] also performed meta-analyses to evaluate the renal protective effect of RIPC in patients undergoing cardiac and vascular interventions, and the results of both showed that serum creatinine levels were not reduced by RIPC. These apparent inconsistencies may be due to limitations in the low number of studies, a small sample size, and different definitions of AKI. Our meta-analysis found that RIPC significantly decreased the incidence of AKI from 11.91% to 6.45% (OR: 0.494; 95% CI: 0.335–0.729; *P* < 0.001), confirming once again that RIPC has a protective effect on renal function in patients undergoing PCI. The causes of renal injury after PCI may include contrast-induced nephropathy and reperfusion injury. The mechanisms of contrast-induced AKI after PCI are still ill-defined and poorly understood, but potential mechanisms underlying CI-AKI include damage to the tubular epithelial cells and vascular endothelium, a change of renal hemodynamics with reduced effective arterial volume during the procedure, microemboli to the kidney, drug toxicity, regional hypoxia, and the production of oxygen free radicals that scavenge nitric oxide (NO) and blunt NO activity [54]. RIPC may promote endothelial oxide synthase to enhance the production of NO and reduce the production of reactive oxygen species, which is an important factor in the late phase of reperfusion, as it reduces damage to the tubular epithelial cells and vascular endothelium [55]. At present, the number of RIPC studies and the sample sizes are still small, so large randomized controlled trials that include a larger number of patients are required to confirm the efficacy of RIPC in AKI in patients undergoing PCI.

The mechanism of RIPC is very complex, but it is mainly concentrated in the mitochondrial ATP-sensitive potassium channel, protein kinase C, and the NF-kappa B molecular mechanism of signal transduction. The inflammatory response is an important mechanism for myocardial ischemia-reperfusion injury [56, 57]. Some studies have indicated that RIPC can protect the myocardium by inhibiting inflammation. In our meta-analysis, we found that RIPC cannot reduce the levels of CRP after PCI. This may be due to the fact that CRP is not a sensitive marker for assessing the inflammatory response of patients undergoing PCI. More sensitive inflammation markers, such as NF-kappa B, IL-6, HMGB1, are worth using to assess the inflammatory status in patients after PCI treatment. Further studies seeking to determine whether RIPC can reduce the inflammatory response after PCI are needed.

## 5. Limitations

Despite the overall robust statistical evidence produced by this analysis, some limitations should be pointed out. First, we were unable to access the individual patient data. The results of the meta-analysis were mainly based on the published merged patient data, such as the mean age, the proportion of males, the proportion of risk factors, and the proportion of various drugs used. Therefore, the effects of RIPC may be underestimated. Second, the RIPC protocol should impact its effects on clinical outcomes; however, we could not determine which protocol was superior to another (e.g., RIPC on arms or legs, different cycle times, etc.). Third, the definition of AKI varied among the individual studies, and this may influence the final incidence of AKI. However, there was no further study on the protective effect of RIPC on renal function in this analysis. Fourth, long-term morbidity and mortality were not evaluated in this meta-analysis because of insufficient data. Lastly, the studies included in this meta-analysis were only publications in English language, which may cause publication bias.

## 6. Conclusion

Our meta-analysis demonstrated that RIPC, using repeated brief episodes of limb ischemia-reperfusion, can provide cardiac and renal protection for patients undergoing elective PCI. RIPC has no beneficial effect on reducing the levels of cTnI and CRP after PCI. Future randomized clinical trials should be performed to apply optimal RIPC protocol and evaluate the long-term clinical outcomes.

## Supplementary Material

Supplementary Figure 1: Begg's funnel plot for publication bias test. (A) the incidence of PMI; (B) the incidence of AKI; (C) the levels of cTnI at 12 h postoperatively; (D) the levels of cTnI at 24 h postoperatively; (E) the levels of CRP 12–24 hours after PCI.
Supplementary Figure 2: Egger's funnel plot for publication bias test. (A) the levels of cTnI at 12 h postoperatively (B) the levels of cTnI at 24 h postoperatively (C) the levels of CRP 12–24 hours after PCI.

## Figures and Tables

**Table 1:** Summarized patients' baseline characteristics of included randomized trials.

Study	Age	Male (%)	DM (%)	HT (%)	Dyslipidemia (%)	Previous MI (%)	Baseline LVEF (%)	Baseline renal function	ACEI (%)	*β*-Blockers (%)	Statins (%)	Multivessel disease (%)	Type C lesion (%)
Ahmed et al.	54	86.6	51.7	63.8	66.4	NA	NA	NA	55	81	72.5	25	15
Carrasco-Chinchilla et al.	65	68.1	42.1	75.6	62.2	NA	58.3	77.2	67.4	82.9	67.5	58.3	NA
Deftereos et al.	68	64	36	65	59	NA	56	75	68	17	36	55.1	NA
Er et al.	73	71	64	91	75	41	59.6	60	NA	82	NA	NA	NA
Ghaemian et al.	61	47.5	36.3	48.8	73.8	8.8	NA	NA	55	81.3	76.3	45	77
Hoole et al.	62	78.2	21.8	51.5	NA	55.4	50.2	NA	74	79.2	95	17	36
Iliodromitis et al.	62	55	34.1	NA	80.5	NA	55	NA	56.1	70.7	61	NA	NA
Lavi et al.	63.7	72.9	32.5	70	67	43	NA	Normal	NA	NA	NA	18.8	NA
Lavi II	64.3	74.2	29.5	70	65	42		Normal	NA	NA	NA	21.7	NA
Liu et al.	58	54.5	36	62.5	NA	NA	61.5	68.33	90.5	81	95.5	54	40.19
Luo et al.	60	76.1	27.8	65.9	NA	21.5	64	100	57	83	NA	28	NA
Melo et al.	NA	NA	NA	NA	NA	NA	NA	NA	NA	NA	NA	NA	NA
Prasad et al.	66	83.2	27.4	77.9	73.7	28.4	56	Normal	38	73	67.4	17	43
Singh et al.	68.9	48	100	85.3	48	7 (6.9)	59.3	47.66	55.9	32.4	80.4	29.4	NA
Xu	69	68	100	63.5	NA	23	63.7	Normal	NA	82.4	100	NA	100
Zografos et al.	61	88	19	82	71.5	20	56.4	88.4	NA	82	96	NA	NA

**Table 2:** Summarized trial design of the included randomized trials.

Study	Year	Country	Number of patients (RIPC/control)	Limb	Protocol of preconditioning	Definition of periproceduralmyocardial infarction	First cuff to balloon time
Ahmed et al.	2013	Egypt	77/72	Arm	200 mmHg × 3 cycles × 5 min	An increase of cTnT greater than 3 times the 99th percentile URL	Several minutes
Carrasco-Chinchilla et al.	2013	Spain	118/114	Arm	200 mmHg × 3 cycles × 5 min	An increase of cTnT greater than 3 times the 99th percentile URL	5 min after PCI
Deftereos et al.	2013	Greece	113/112	Arm	200 mmHg × 4 cycles × 30 sec	NA	Several minutes before PCI
Er et al.	2012	Germany	26/26	Arm	50 mmHg > SBP × 4 cycles × 5 min	NA	40–85 min
Ghaemian et al.	2012	Iran	40/40	Leg	>SBP × 2 cycles × 5 min	An increase of cTnT greater than 3 times the 99th percentile URL	65 min
Hoole et al.	2009	UK	126/125	Arm	200 mmHg × 3 cycles × 3 min	An increase of cTnT greater than 3 times the 99th percentile URL	96 min
Iliodromitis et al.	2006	Greece	20/21	Arm	200 mmHg × 3 cycles × 3 min	NA	30 min
Lavi et al.	2014	Canada	120/120	Arm	200 mmHg or 50 mmHg > SBP × 1 cycle × 5 min	An increase of cTnT greater than 5 times the 99th percentile URL	Several minutes after PCI
Lavi II	2014	Canada	120/120	Leg	200 mmHg or 50 mmHg > SBP × 1 cycle × 5 min	An increase of cTnT greater than 5 times the 99th percentile URL	Several minutes after PCI
Liu et al.	2014	China	98/102	Arm	200 mmHg × 3 cycles × 5 min	NA	18–24 hours
Luo et al.	2013	China	101/104	Arm	200 mmHg × 3 cycles × 5 min	An increase of cTnT greater than 5 times the 99th percentile URL	<120 min
Melo et al.	2013	Brazil	9/20	Arm	200 mmHg × 3 cycles × 5 min	An increase of cTnT greater than 3 times the 99th percentile URL	NA
Prasad et al.	2013	USA	47/48	Arm	200 mmHg × 3 cycles × 3 min	An increase of cTnT greater than 3 times the 99th percentile URL	>18 min
Singh et al.	2016	Korea	51/51	Arm	200 mmHg × 3 cycles × 5 min	NA	30 min
Xu	2013	China	102/98	Arm	200 mmHg × 3 cycles × 5 min	An increase of cTnT greater than 3 times the 99th percentile URL	30–120 min
Zografos et al.	2014	Greece	47/47	Arm	200 mmHg × 1 cycle × 5 min	An increase of cTnT greater than 5 times the 99th percentile URL	4 min

**Table 3:** Jadad et al.'s scores of included studies.

Study	Randomization	Double-blinding	Withdrawals	Randomization methods	Double-blinding methods	Total score
Ahmed et al., 2013	1	0	1	0	0	2
Carrasco-Chinchilla et al., 2013	1	1	1	0	0	3
Deftereos et al.	1	0	1	1	0	5
Er et al., 2012	1	1	1	1	1	5
Ghaemian et al., 2012	1	1	1	1	1	4
Hoole et al., 2009	1	1	1	1	1	5
Iliodromitis et al., 2006	1	0	1	0	0	2
Lavi et al., 2014	1	1	1	1	1	5
Lavi II, 2014	1	1	1	1	1	5
Liu et al., 2014	1	0	1	0	0	3
Luo et al., 2013	1	0	1	0	0	3
Melo et al., 2013	N.A	N.A	N.A	N.A	N.A	N.A
Prasad et al., 2013	1	0	1	0	0	2
Singh et al., 2016	1	1	1	1	1	5
Xu, 2013	1	1	1	0	0	5
Zografos et al., 2014	1	1	1	0	1	3
