# Supplementary material for: Effect of Remote Ischemic Preconditioning on Perioperative Cardiac Events in Patients Undergoing Elective Percutaneous Coronary Intervention: A Meta-Analysis of 16 Randomized Trials
Source: Cardiol Res Pract. 2017 Sep 14;2017:6907167. doi: 10.1155/2017/6907167 (PMC5618784; doi:10.1155/2017/6907167)
Supplement: Supplementary file 1 — Supplementary Figure 1: Begg's funnel plot for publication bias test. (A) the incidence of PMI; (B) the incidence of AKI; (C) the levels of cTnI at 12 h postoperatively; (D) the levels of cTnI at 24 h postoperatively; (E) the levels of CRP 12–24 hours after PCI. Supplementary Figure 2: Egger's funnel plot for publication bias test. (A) the levels of cTnI at 12 h postoperatively (B) the levels of cTnI at 24 h postoperatively (C) the levels of CRP 12–24 hours after PCI. [file 6907167.f1.pdf]

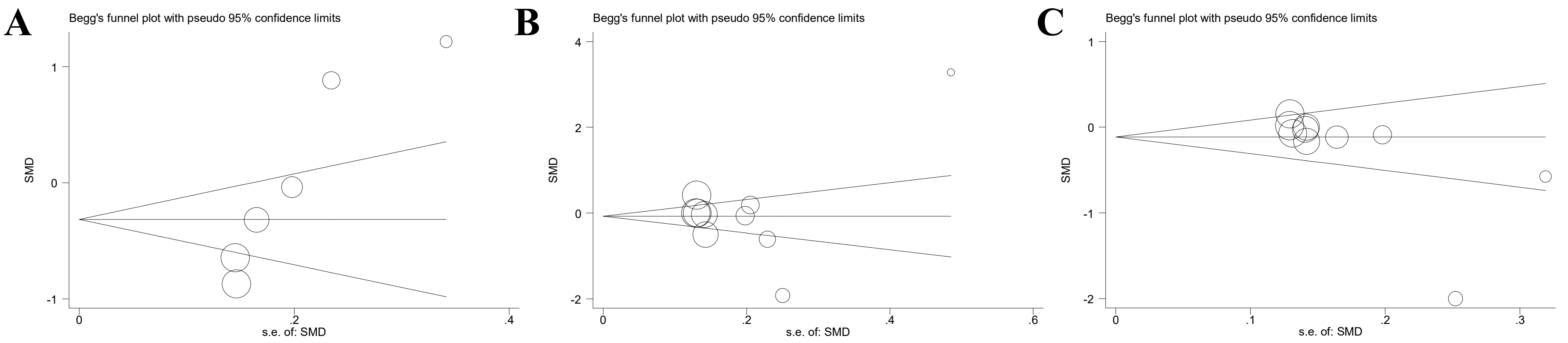

Supplementary Figure 1 The Begg's funnel plot for publication bias test. **(A)** the incidence of PMI; **(B)** the incidence of AKI; **(C)** the levels of cTnI at 12h postoperatively; **(D)** the levels of cTnI at 24h postoperatively; **(E)** the levels of CRP 12-24 hours after PCI.

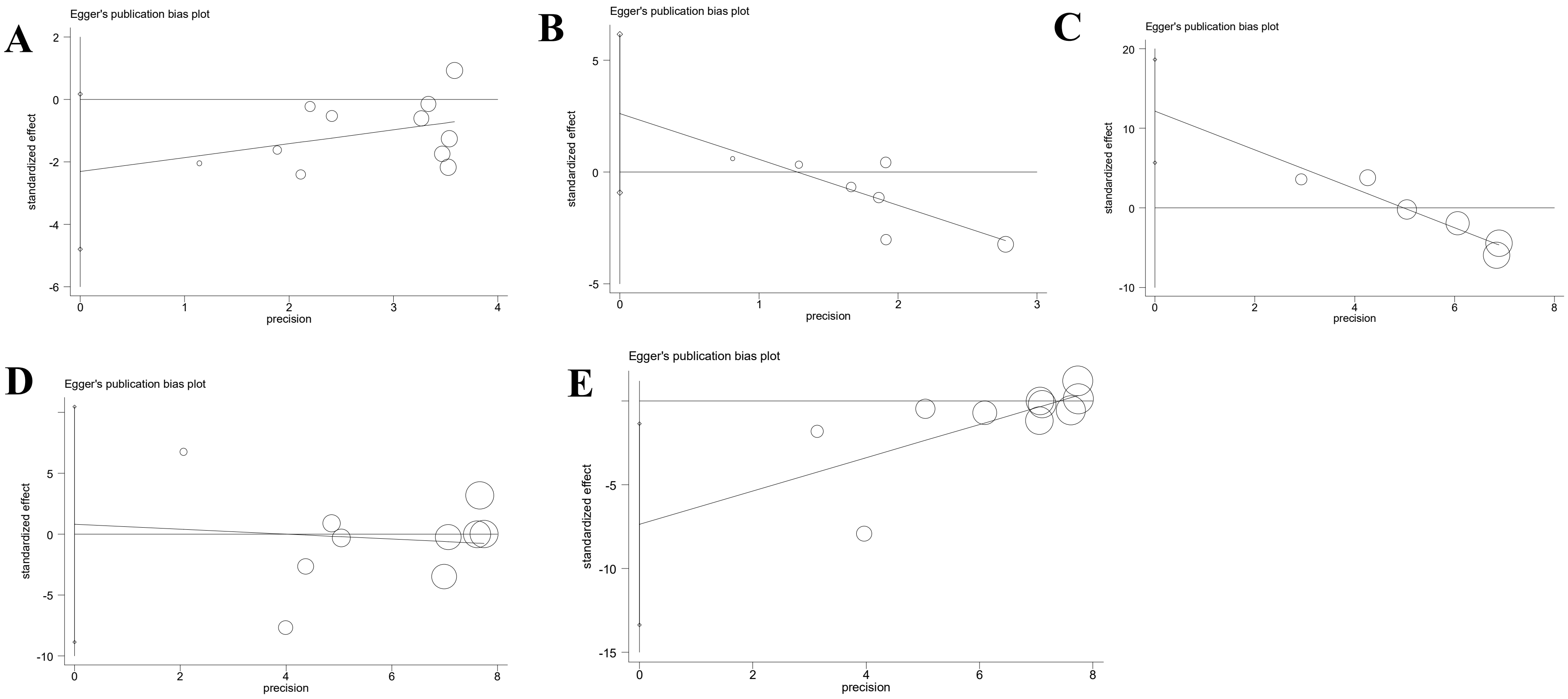

Supplementary Figure 2 The egger's funnel plot for publication bias test. **(A)** the levels of cTnI at 12h postoperatively **(B)** the levels of cTnI at 24h postoperatively **(C)** the levels of CRP 12-24 hours after PCI. cTnI: Troponin I; CPR:C-reactive protein.
